# F-ATP synthase inhibitory factor 1 regulates metabolic reprogramming involving its interaction with c-Myc and PGC1α

**DOI:** 10.3389/fonc.2023.1207603

**Published:** 2023-07-03

**Authors:** Lishu Guo, Zhenglong Gu

**Affiliations:** ^1^ Center for Mitochondrial Genetics and Health, Greater Bay Area Institute of Precision Medicine (Guangzhou), Fudan University, Guangzhou, China; ^2^ Tongji University Cancer Center, Shanghai Tenth People’s Hospital, School of Medicine, Tongji University, Shanghai, China

**Keywords:** mitochondria, F-ATP synthase inhibitory factor 1, metabolic reprogramming, c-Myc, p-c-Myc, PGC1α

## Abstract

F-ATP synthase inhibitory factor 1 (IF1) is an intrinsic inhibitor of F-ATP synthase. It is known that IF1 mediates metabolic phenotypes and cell fate, yet the molecular mechanisms through which IF1 fulfills its physiological functions are not fully understood. Ablation of IF1 favors metabolic switch to oxidative metabolism from glycolysis. c-Myc and PGC1α are critical for metabolic reprogramming. This work identified that IF1 interacted with Thr-58 phosphorylated c-Myc, which might thus mediate the activity of c-Myc and promote glycolysis. The interaction of IF1 with PGC1α inhibited oxidative respiration. c-Myc and PGC1α were localized to mitochondria under mitochondrial stress in an IF1-dependent manner. Furthermore, IF1 was found to be required for the protective effect of hypoxia on c-Myc- and PGC1α-induced cell death. This study suggested that the interactions of IF1 with transcription factors c-Myc and PGC1α might be involved in IF1-regulatory metabolic reprogramming and cell fate.

## Introduction

F-ATP synthase inhibitory factor 1, encoded by ATP5IF1 gene, is an intrinsic inhibitor of F-ATP synthase (1). The binding of IF1 to F-ATP synthase depends on matrix pH (2–4) and its phosphorylation by PKA (5). IF1 acts as a regulator of mitochondrial biogenesis and physiology (6–8). IF1 expression varies between different tissues and cell lines, and mediates heterogeneous metabolic phenotypes and cell fate (6, 9, 10). IF1 inhibits mitochondrial oxidative phosphorylation (OXPHOS) and enhances glycolytic activity, promoting metabolic reprogramming to a Warburg phenotype (11). IF1 preserves mitochondrial bioenergetics during hypoxia by preventing ATP hydrolysis (12). IF1 promotes mitochondrial depolarization during uncoupling and is essential to trigger mitophagy (13). IF1 regulates heme synthesis via modulation of mitochondrial pH and redox potential (14). IF1 also modulates angiogenesis by its ability to conserve ATP on the endothelial cell surface (15).

c-Myc is a global amplifier of gene expression and has been implicated in various cellular processes including cell proliferation, differentiation, apoptosis, and metabolism (16). c-Myc binds to open chromatin and stimulates transcription (17). c-Myc can access many metabolic genes and genes associated with mitochondrial function, which contributes to metabolic reprogramming (17). Metabolic alterations induced by c-Myc activate AMPK, a sensor of cellular energetic status that is signaled by intracellular ADP/ATP and AMP/ATP ratios (18). c-Myc augments nuclear-encoded mitochondrial gene expression and promotes mitochondrial respiration, which directly facilitates generation of mitochondrial reactive oxygen species (ROS) and, thus, genomic instability (19). The oncogene is deregulated and contributes broadly to human cancers, but is strictly regulated in normal cells (17, 20). c-Myc targets several mitochondrial genes and regulates mitochondrial biogenesis (21). C-Myc extracts induce mitochondrial outer membrane permeabilization and cytochrome *c* release from purified mouse liver mitochondria in a Bid-dependent manner (22), indicating that c-Myc may interact with mitochondrial proteins and regulate mitochondrial function.

The peroxisome proliferator-activated receptor gamma coactivator 1-alpha (PGC1α) is a member of the PGC-1 family and synchronizes the mitochondrial and nuclear genomes (23). PGC1α coordinates mitochondrial biogenesis including synthesis of mitochondrial proteins and phospholipids as well as mitochondrial DNA replication (23, 24). Activation of PGC1α stimulates mitochondrial oxidative metabolism through specific bindings to various transcription factors (25). Reprogramming of energy metabolism is one of the hallmarks of cancer cells (26). PGC1α is a critical regulator of cancer progression by maintenance of metabolic balance and facilitating chemoresistance (27). The tumor suppressor p53 mediates ROS clearance, cell-cycle arrest, apoptosis, and mitochondrial metabolism (27, 28). PGC1α binds to p53 and regulates p53 transactivation of cell-cycle arrest and metabolic genes (28). PGC1α can be present inside mitochondria, and this mitochondrial counterpart may mediate the cross-talk between cellular metabolism and mitochondrial biogenesis (29).

Although it is known that IF1 mediates metabolic phenotypes and cell fate, how does IF1 fulfill its physiological functions is not fully understood. Nuclear transcription factors can reside in mitochondria and regulate mitochondrial function through interaction with mitochondrial proteins (30–34). c-Myc and PGC1α are critical for metabolic reprogramming and cell fate decision. The balance of c-Myc and PGC1α determines the metabolic plasticity of pancreatic cancer stem cells (35). However, whether c-Myc and PGC1α could localize to mitochondria and interact with IF1 to regulate metabolic reprogramming remains to be investigated. This work showed that IF1 bound to c-Myc and Thr-58 phosphorylated c-Myc as well as PGC1α in mitochondria. These bindings may promote degradation of c-Myc and destabilize PGC1α in mitochondria, which mediates metabolic reprogramming.

## Results

### Ablation of IF1 promotes metabolic reprogramming to OXPHOS

**Figure 1:**
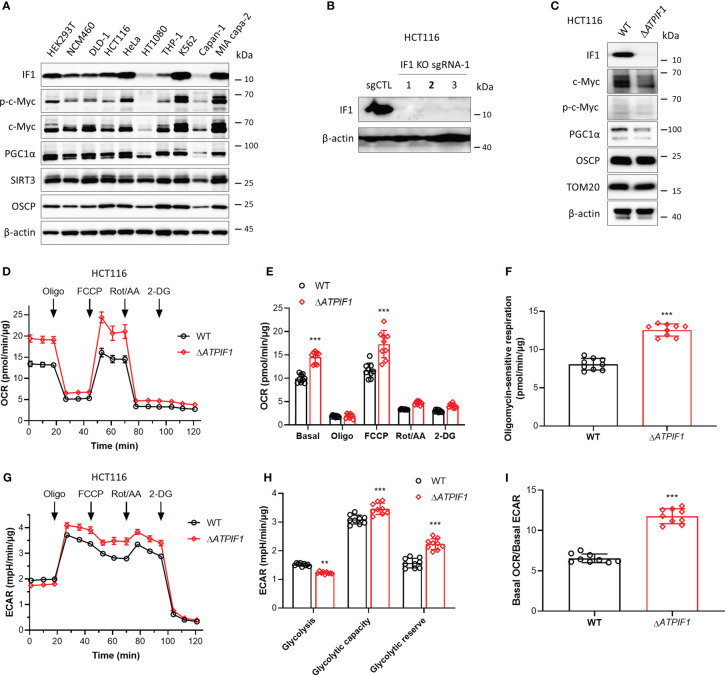
Ablation of IF1 promotes metabolic reprogramming to OXPHOS. **(A)** Representative blots of protein extracts of indicated cell lines analyzed by Western blotting (WB). **(B)** Expression of IF1 in HCT116 clonal cells after disruption of *ATPIF1* gene with sgRNA-1 using the CRISPR/Cas9 technique. Colony 2 (number in bold) was selected for the following experiments. **(C)** Cellular protein extracts were analyzed by WB. OXPHOS **(D–I)** and glycolysis **(G–I)** activities were evaluated by Agilent Seahorse XFe24 Analyzer before and after additions of oligomycin (Oligo, 2 µM), FCCP (0.25 µM), rotenone plus antimycin A (Rot/AA, 1 µM), and 2-DG (50 mM). OCR values (pmol/min) were normalized for protein (µg). **(D)** *R*epresentative traces of OCR values (pmol/min/µg) in wild-type (WT, black trace) and IF1 KO (Δ*ATPIF1*, red trace). **(E)** OCR values (pmol/min/µg) in WT (black column) and Δ*ATPIF1* (red column). In groups of Basal, Oligo, and FCCP, the OCR values were subtracted for Rot/AA. Data are expressed as mean ± SD. ^***^ *p* < 0.001 *vs.* WT, two-way ANOVA with Bonferroni *post-hoc* test. **(F)** Oligomycin-sensitive respiration was expressed as mean ± SD. ^***^ *p* < 0.001 *vs.* WT, one-way ANOVA with Bonferroni *post-hoc* test. **(G, H)** ECAR values (mpH/min) were normalized for protein (µg). **(G)** Representative traces of ECAR values (mpH/min/µg) in wild-type (WT, black trace) and IF1 KO (Δ*ATPIF1*, red trace). **(H)** ECAR values (mpH/min/µg) were subtracted for 2-DG and expressed as mean ± SD. ^**^ *p* < 0.01 *vs.* WT, ^***^ *p* < 0.001 *vs.* WT, two-way ANOVA with Bonferroni *post-hoc* test. **(I)** Ratio of basal OCR value and basal ECAR value. ^***^ *p* < 0.001 *vs.* WT, one-way ANOVA with Bonferroni *post-hoc* test.

IF1 plays a role in mediating metabolic reprogramming and is upregulated in many cancers contributing to Warburg phenotype (11). IF1 expression varies in different tissues and cell types (6) (**Figure 1A**). The phosphorylation level of c-Myc on the Thr-58 site appeared to be positively correlated with the expression level of IF1 (**Figure 1A**). In addition, the expression levels of SIRT3 and OSCP were both upregulated in some cell lines including HCT116 and MIA PaCa-2 cells (**Figure 1A**), which contributed to cell adaption to mitochondrial stress. The CRISPR/Cas9 technique was used to disrupt *ATPIF1* gene in HCT116 cells (**Figure 1B**). The ablation of IF1 led to the declined expressions of c-Myc and PGC1α (**Figure 1C**), indicating that IF1 might mediate metabolic reprogramming through interplay with these transcription factors. The influence of *ATPIF1* inactivation on metabolic phenotype was detected by Seahorse XF Analyzer. The ablation of IF1 led to a significant rise in both basal and maximal oxidative respiration (**Figures 1D, E**), as well as oligomycin-sensitive mitochondrial respiration (**Figure 1F**). The basal glycolytic metabolism was inhibited by ablation of IF1 (**Figures 1G, H**), suggesting that disruption of *ATPIF1* gene favors metabolic switch to OXPHOS from glycolysis (**Figure 1I**). Interestingly, the ablation of IF1 caused significantly increased glycolytic capacity and glycolytic reserve (**Figures 1G, H**), indicating that IF1 plays a role in mediating glycolytic metabolism.

### IF1 participates in enhanced glycolysis driven by c-Myc

**Figure 2:**
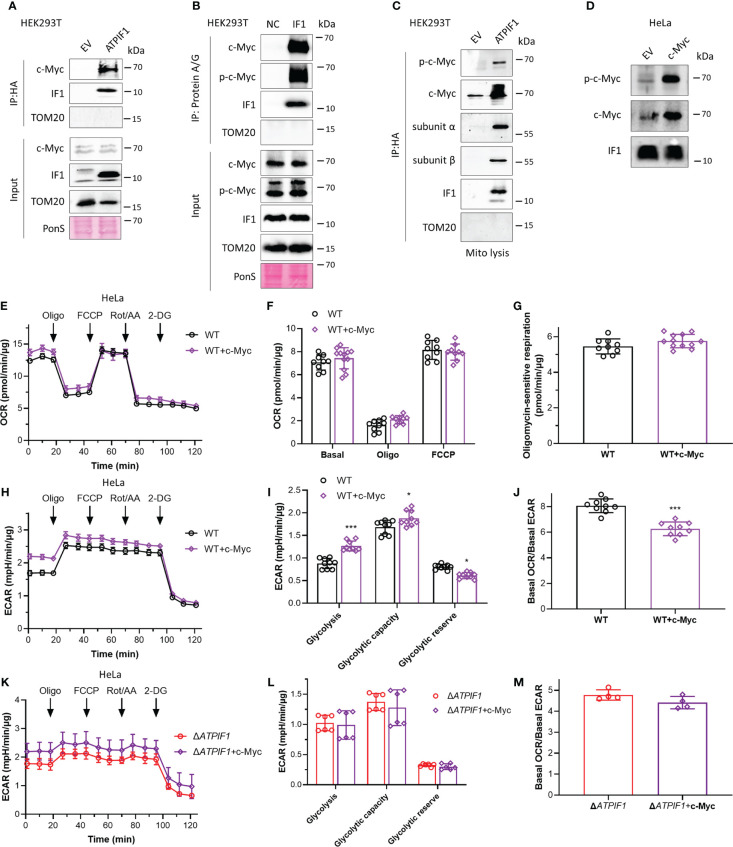
IF1 participates in enhanced glycolysis driven by c-Myc. **(A, C)** HEK293T cells were transfected with empty vector (EV) or plasmids carrying *ATPIF1* and incubated for 24 h. In **(A)**, cells were lysed for coIP and WB. In panel C, isolated mitochondria (mito) were lysed for coIP and WB. **(B)** HEK293T cells were lysed for coIP and WB. In negative control (NC), only Protein A/G Plus Agarose Beads and ATPIF1 antibody were incubated with coIP buffer at 4°C overnight. In IF1, Protein A/G Plus Agarose Beads and ATPIF1 antibody were incubated with cell lysate at 4°C overnight. PonS indicated the ponceau S staining of transferred membraned. **(D–J)** WT HeLa cells were transfected with EV or plasmids carrying *c-Myc* and incubated for 24 h. **(D)** Cells were collected from XF24 Cell Culture Microplates after Seahorse experiment and lysed for WB. OXPHOS **(E–J)** and glycolysis **(H–J)** activities were evaluated by Agilent Seahorse XFe24 Analyzer before and after the addition of oligomycin (Oligo, 2 µM), FCCP (0.25 µM), rotenone plus antimycin A (Rot/AA, 1 µM), and 2-DG (50 mM). OCR values (pmol/min) were normalized for protein (µg). **(E)** Representative traces of OCR values (pmol/min/µg) in HeLa cells transfected with EV (black trace) and plasmids carrying *c-Myc* (purple trace). **(F)** OCR values (pmol/min/µg) were subtracted for Rot/AA and expressed as mean ± SD. **(G)** Oligomycin-sensitive respiration was expressed as mean ± SD. **(H, I)** ECAR values (mpH/min) were normalized for protein (µg). **(H)** Representative traces of ECAR values (mpH/min/µg) in WT HeLa cells transfected with EV (black trace) and plasmids carrying *c-Myc* (purple trace). **(I)** ECAR values (mpH/min/µg) were subtracted for 2-DG and expressed as mean ± SD. ^*^ *p* < 0.05 *vs.* EV, ^***^ *p* < 0.001 *vs.* EV, two-way ANOVA with Bonferroni *post-hoc* test. **(J)** Ratio of basal OCR value and basal ECAR value in WT HeLa cells. ^***^ *p* < 0.001 *vs.* EV, one-way ANOVA with Bonferroni *post-hoc* test. **(K–M)** Glycolysis activities of Δ*ATPIF1* HeLa cells. **(K, L)** ECAR values (mpH/min) were normalized for protein (µg). **(K)** Representative traces of ECAR values (mpH/min/µg) in Δ*ATPIF1* HeLa cells transfected with EV (red trace) and plasmids carrying *c-Myc* (violet trace). **(L)** ECAR values (mpH/min/µg) were subtracted for 2-DG and expressed as mean ± SD. **(M)** Ratio of basal OCR value and basal ECAR value in Δ*ATPIF1* HeLa cells.

c-Myc drives metabolic reprogramming, which favors tumorigenesis and cancer cell survival (36). c-Myc enhances glycolytic gene expression and activation of c-Myc drives aerobic glycolysis, thus contributing to oncogenic metabolic state (36, 37). Phosphorylation sites Thr-58 and Ser-62 control c-Myc-dependent transactivation of gene expression and regulate c-Myc protein stability (38–40). Decreased Thr-58 and increased Ser-62 phosphorylation stabilize c-Myc protein, and ratios of Thr-58 and Ser-62 phosphorylation are altered in human cancer (39). Immunoprecipitation assay revealed that IF1 strongly bound to c-Myc (**Figure 2A**) and Thr-58 phosphorylated c-Myc (**Figures 2A, B**). To further investigate whether this binding could occur in mitochondria, mitochondria were purified for immunoprecipitation assay. IF1 bound to both c-Myc and Thr-58 phosphorylated c-Myc in mitochondria (**Figure 2C**). Overexpression of c-Myc accompanied by enhanced phosphorylation of c-Myc on the Thr-58 site (**Figure 2D**) did not affect oxidative metabolism (**Figures 2E–G**) but promoted glycolysis (**Figures 2H–J**). The ablation of IF1 abolished the stimulating effect of c-Myc on glycolytic activity (**Figures 2K–M**). The observations suggested that IF1 bound to c-Myc in mitochondria and then mediated the phosphorylation and activity of c-Myc.

### High glucose promotes cellular metabolism involving stimulation of c-Myc localization to mitochondria and its interactions with IF1

**Figure 3:**
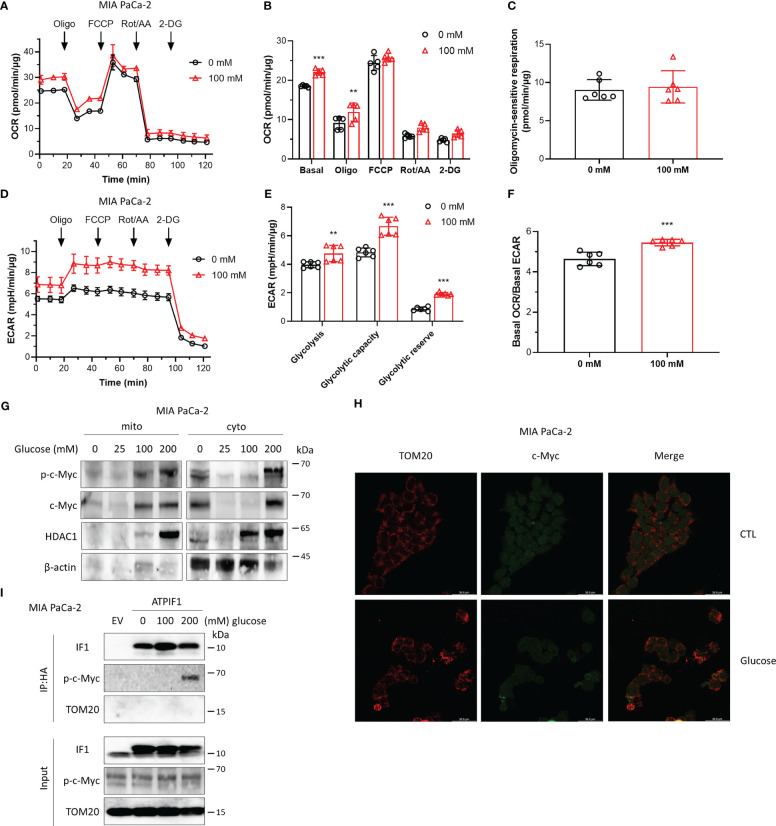
High glucose promotes cellular metabolism involving stimulation of c-Myc localization to mitochondria and its interactions with IF1. **(A–F)** OXPHOS **(A–C, F)** and glycolysis **(D–F)** activities were evaluated by Agilent Seahorse XFe24 Analyzer before and after additions of oligomycin (Oligo, 2 µM), FCCP (0.2 µM), rotenone plus antimycin A (Rot/AA, 1 µM), and 2-DG (50 mM). MIA PaCa-2 cells were suspended in DMEM (no sodium pyruvate, no glucose, Gibco #11966025) supplemented with 10% FBS and seeded in XF24 microplates at 1.6×10^4^ cells/well, then incubated at 37°C in a 5% CO_2_ humidified incubator 24 h later, and cells were treated with indicated concentrations of glucose for 48 h. OCR values (pmol/min) and ECAR values (mpH/min) were normalized for protein (µg). **(A)** Representative traces of OCR values (pmol/min/µg) of MIA PaCa-2 cells cultured in DMEM medium without glucose (black trace) and in DMEM medium with 100 mM glucose (red trace). **(B)** OCR values (pmol/min) were normalized for protein (µg) and expressed as OCR (pmol/min/µg). In groups of Basal, Oligo, and FCCP, the OCR values were subtracted for Rot/AA. Data are expressed as mean ± SD. ^**^ *p* < 0.01 *vs.* 0 mM, ^***^ *p* < 0.001 *vs.* 0 mM, two-way ANOVA with Bonferroni *post-hoc* test. **(C)** oligomycin-sensitive respiration was expressed as mean ± SD. **(D–F)** ECAR values (mpH/min) were normalized for protein (µg). **(D)** Representative traces of ECAR values (mpH/min/µg) of MIA PaCa-2 cells cultured in DMEM medium without glucose (black trace) and in DMEM medium with 100 mM glucose (red trace). **(E)** ECAR values (mpH/min/µg) were subtracted for 2-DG and expressed as mean ± SD. ^**^ *p* < 0.01 *vs.* 0 mM, ^***^ *p* < 0.001 *vs.* 0 mM, two-way ANOVA with Bonferroni *post-hoc* test. **(F)** Ratio of basal OCR value and basal ECAR value. ^***^ *p* < 0.001 *vs.* 0 mM, one-way ANOVA with Bonferroni *post-hoc* test. **(G–I)** MIA PaCa-2 cells were cultured in DMEM (no sodium pyruvate, no glucose, Gibco #11966025) supplemented with 10% FBS. **(G)** Representative blots of protein extracts of isolated mitochondria (mito) and cytosolic fraction (cyto) from MIA PaCa-2 cells treated by indicated concentrations of glucose for 48 h. **(H)** Representative immunofluorescence images (scale bar: 36.8 µm) of MIA PaCa-2 cells treated without (CTL) or with 200 mM glucose for 48 h. Cells were stained with anti-TOM20 (red) and anti-c-Myc (green). **(I)** MIA PaCa-2 cells were treated by indicated concentrations of glucose for 48 h and transfected with EV or plasmids carrying *ATPIF1* for 24 h. Cells were collected and lysed for coIP and WB. The blots are representative of three independent experiments.

Cancer cells are highly dependent on glucose to fuel their energy demand and proliferate, and c-Myc plays a key role in aerobic glycolysis. The effects of high glucose on cellular metabolism and the interaction between IF1 and c-Myc were then investigated. Exposure to high glucose promoted both oxidative and glycolytic activities (**Figures 3A–E**), while oxidative respiration was more influenced with a significant increase of basal OCR/basal ECAR ratio (**Figure 3F**). The maximal oxygen consumption rate induced by injection of FCCP and oligomycin sensitive respiration were almost identical in the absence or presence of glucose (**Figures 3A–C**), indicating that mitochondrial integrity and function were not affected by the pre-exposure to high glucose. The glycolytic capacity and reserve were significantly enhanced by high glucose (**Figures 3D, E**). High glucose enhanced the expressions of c-Myc and Thr-58 phosphorylated c-Myc, and their translocations to mitochondria (**Figure 3G**). Consistent with the observation of Western blotting analysis, immunofluorescence staining revealed that a fraction of c-Myc was localized to mitochondria upon high glucose stimulation (**Figure 3H**), which was required for the interaction with IF1 and Thr-58 phosphorylation of c-Myc (**Figure 3I**). These observations suggest that the localization of c-Myc to mitochondria and its interaction with IF1 might promote cellular metabolism and metabolic reprogramming.

### IF1 binds to PGC1α and inhibits mitochondrial oxidative respiration

**Figure 4:**
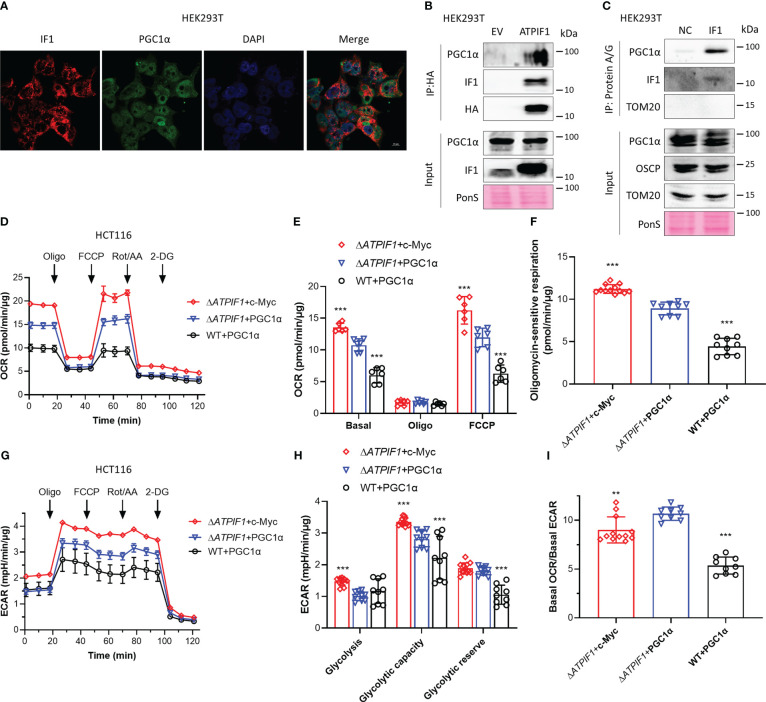
IF1 binds to PGC1α and inhibits mitochondrial oxidative respiration. **(A)** Representative immunofluorescence images (scale bar: 10 µm) of HEK293T cells stained with anti-IF1 (red) and anti-PGC1α (green), and co-labeled with DAPI (blue). **(B)** HEK293T cells were transfected with EV or plasmids carrying *ATPIF1* and incubated for 24 h. Cells were lysed for coIP and WB. **(C)** HEK293T cells were lysed for coIP and WB. In NC, only Protein A/G Plus Agarose Beads and ATPIF1 antibody were incubated with coIP buffer at 4°C overnight. In IF1, Protein A/G Plus Agarose Beads and ATPIF1 antibody were incubated with cell lysate at 4°C overnight. PonS indicated the ponceau S staining of transferred membranes. **(D–I)** Δ*ATPIF1* HCT116 cells were transfected with plasmids carrying *c-Myc* (Δ*ATPIF1*+c-Myc) or *PPARGC1A* (encoding PGC1α) (Δ*ATPIF1*+PGC1α), and WT HCT116 cells were transfected with plasmid carrying *PPARGC1A* (WT+PGC1α), then co-cultured for 24 h. OXPHOS **(D–I)** and glycolysis **(G–I)** activities were evaluated by Agilent Seahorse XFe24 Analyzer before and after the addition of oligomycin (Oligo, 2 µM), FCCP (0.25 µM), rotenone plus antimycin A (Rot/AA, 1 µM), and 2-DG (50 mM). OCR values (pmol/min) were normalized for protein (µg). **(D)** Representative traces of OCR values (pmol/min/µg) in Δ*ATPIF1*+c-Myc (purple trace), Δ*ATPIF1*+PGC1α (blue trace), and WT+PGC1α (gray trace). **(E)** OCR values (pmol/min/µg) were subtracted for Rot/AA and expressed as mean ± SD. ^***^ *p* < 0.001 *vs.* Δ*ATPIF1*+PGC1α, two-way ANOVA with Bonferroni *post-hoc* test. **(F)** oligomycin-sensitive respiration was expressed as mean ± SD. ^***^ *p* < 0.001 *vs.* Δ*ATPIF1*+PGC1α, one-way ANOVA with Bonferroni *post-hoc* test. **(G, H)** ECAR values (mpH/min) were normalized for protein (µg). **(G)** Representative traces of ECAR values (mpH/min/µg) in Δ*ATPIF1*+c-Myc (purple trace), Δ*ATPIF1*+PGC1α (blue trace), and WT+PGC1α (gray trace). **(H)** ECAR values (mpH/min/µg) were subtracted for 2-DG and expressed as mean ± SD. ^***^ *p* < 0.001 *vs.* Δ*ATPIF1*+PGC1α, two-way ANOVA with Bonferroni *post-hoc* test. **(I)** Ratio of basal OCR value and basal ECAR value. ^**^ *p* < 0.01 *vs.* Δ*ATPIF1*+PGC1α, ^***^ *p* < 0.001 *vs.* Δ*ATPIF1*+PGC1α, one-way ANOVA with Bonferroni *post-hoc* test.

Activation of PGC1α promotes mitochondrial oxidative respiration (25), and the c-Myc/PGC1α ratio is a main controller of metabolic phenotypes and plasticity in pancreatic cancer stem cells (27). IF1 is a mitochondrial specific protein (33). Immunofluorescence analysis showed that PGC1α could localize to both nucleus and mitochondria (**Figure 4A**). Immunoprecipitation revealed that IF1 could bind to PGC1α (**Figures 4B, C** **)**. In the absence of IF1, c-Myc exhibited more significant enhancement on mitochondrial oxidative respiration and glycolytic metabolism than PGC1α (**Figures 4D–H**). However, the effect of c-Myc on glycolytic metabolism was more significant than that on oxidative metabolism, resulting in a significant decrease of OCR/ECAR ratio compared to the effect of PGC1α (**Figure 4I**). The presence of IF1 led to a dramatic decline of oxidative metabolism but did not influence the glycolytic metabolism (**Figures 4D–I**). IF1 might interact with PGC1α and destabilize PGC1α, resulting in a significant decrease of mitochondrial oxidative phosphorylation. The stability of c-Myc and PGC1α inhibited by IF1 may contribute to metabolic reprogramming.

### The presence of IF1 is required for c-Myc and PGC1α imported into mitochondria under mitochondrial stress

**Figure 5:**
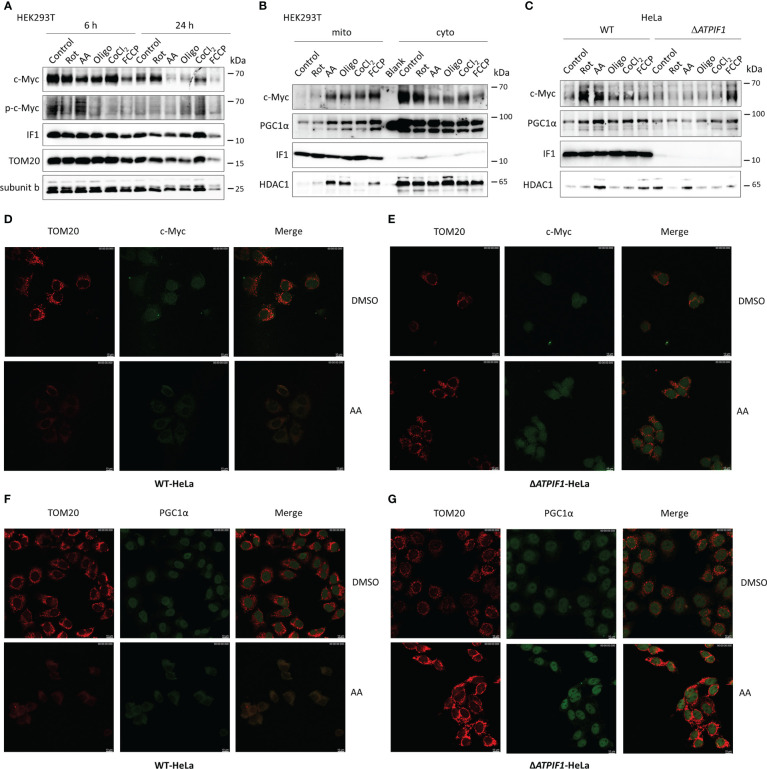
The presence of IF1 is required for c-Myc and PGC1α imported into mitochondria under mitochondrial stress. **(A)** Representative blots of protein extracts of HEK293T cells treated by equivalent DMSO (Control), 10 µM rotenone (Rot), 10 µM antimycin A (AA), 10 µM oligomycin (Oligo), 0.2 mM CoCl_2_, and 10 µM FCCP for 6 h or 24 h. The blots are representative of three independent experiments. **(B)** Representative blots of protein extracts of isolated mitochondria (mito) and cytosolic fraction (cyto) from HEK293T cells treated by Control, Rot, AA, Oligo, CoCl_2_, and FCCP for 6 h. The blots are representative of four independent experiments. **(C)** Representative blots of protein extracts of isolated mitochondria from WT and Δ*ATPIF1* HeLa cells treated by Control, Rot, AA, Oligo, CoCl_2_, and FCCP for 6 h. The blots are representative of five independent experiments. **(D–G)** Representative immunofluorescence images (scale bar: 10 µm) of WT **(D, F)** and Δ*ATPIF1* **(E, G)** HeLa cells treated by DMSO or AA for 6 h. Cells were stained with anti-TOM20 (red) and anti-c-Myc (green) or anti-PGC1α (green).

The role of IF1 in c-Myc and PGC1α imported into mitochondria was further investigated. Inhibition of mitochondrial respiration or induction of mitochondrial dysfunction slightly reduced the expression levels of c-Myc and PGC1α (**Figure 5A**). The longer-term exposure to mitochondrial inhibitors induced a dramatic decrease in mitochondrial mass, which could be caused by mitophagy, resulting in a striking decline of c-Myc and PGC1α expressions (**Figure 5A**). Of note, mitochondrial stress induced by inhibitors of mitochondrial respiration, CoCl_2_, or uncoupler FCCP promoted c-Myc and PGC1α import into mitochondria (**Figures 5B, D, F**). The ablation of IF1 prevented the mitochondrial import of c-Myc and PGC1α under mitochondrial stress, for instance, after antimycin A treatment (**Figures 5C, E, G**). These observations indicated that IF1 was required for the mitochondrial import of c-Myc and PGC1α under mitochondrial stress condition.

### IF1 is required for the protective effect of hypoxia on c-Myc/PGC1α-induced cell death

The combination with c-Myc stimulation of abnormal mitochondria and hypoxic microenvironment in tumors synergistically stimulates mitochondrial ROS production through the ineffective function of respiratory complex III under limiting oxygen conditions (19, 41). Chemical incubation cells with CoCl_2_ induce cellular hypoxia, which triggers dephosphorylation of IF1 and inhibits both the synthetic and hydrolytic activities of F-ATP synthase (5). Hypoxic microenvironment and glucose deprivation facilitate proteolytic degradation of c-Myc and attenuate its function (36, 42). Hypoxia stimulates the expression of PGC1α and mitochondrial biogenesis in cardiac myocytes (43). Mild ROS intensity triggers nuclear reprogramming, which is involved in the metabolic adaption and activation of cellular survival (7).

**Figure 6:**
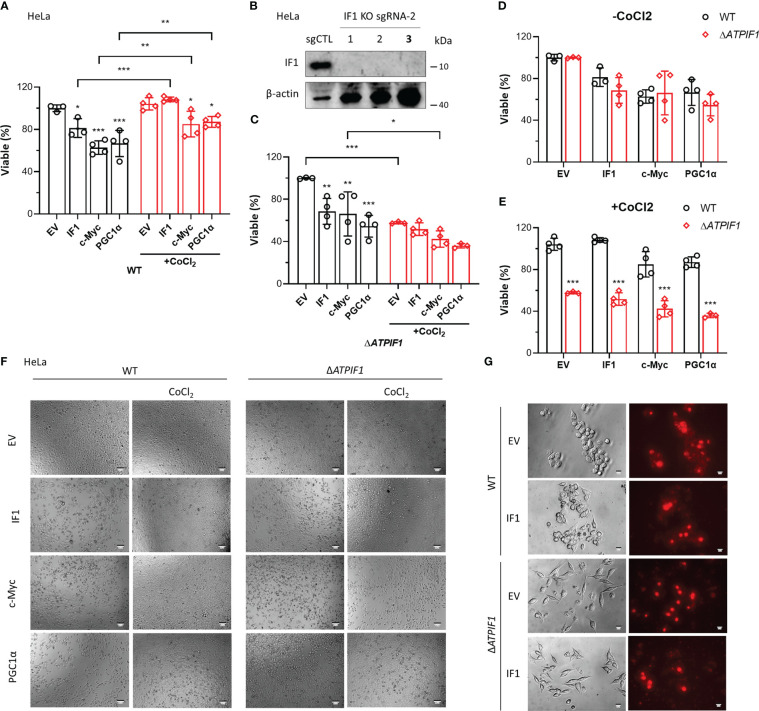
IF1 is required for the protective effect of hypoxia on c-Myc/PGC1α-induced cell death. **(A, C–E)** The effects of IF1/c-Myc/PGC1α overexpression and CoCl_2_-induced hypoxic environment on cell viability in WT and Δ*ATPIF1* HeLa cells revealed by MTT assay. Viable (%) was expressed as mean ± SD. ^*^ *p* < 0.05, ^**^ *p* < 0.01, ^***^ *p* < 0.001 *vs.* EV or WT, two-way ANOVA with Bonferroni *post-hoc* test. **(F)** The images of WT and Δ*ATPIF1* HeLa cells with overexpression of IF1/c-Myc/PGC1α and CoCl_2_ treatment for 24 h. The figures are representative of at least three independent experiments. **(B)** Expression of IF1 in HeLa clonal cells after disruption of *ATPIF1* gene with sgRNA-2 using the CRISPR/Cas9 technique. Colony 3 (number in bold) was used for MTT assay as shown in **(A, C–F)**. **(G)** HCT116 WT and IF1 KO cells were transfected with EV or plasmids carrying *ATPIF1* for IF1 overexpression (IF1 OE), then incubated for 24 h, followed by MitoSOX staining.

Overexpression of IF1, c-Myc, or PGC1α induced cell death, which was prevented by CoCl_2_ (**Figures 6A, F**). The ablation of IF1 (**Figure 6B**) abolished the protective function of CoCl_2_ in IF1/c-Myc/PGC1α overexpression-induced cell death (**Figures 6C, F**). Knockout of IF1 had no effect on IF1/c-Myc/PGC1α overexpression-induced cell death, unless CoCl_2_ was added (**Figures 6D–F**). These results suggested that IF1 was necessary for the protective role of hypoxia in c-Myc/PGC1α-induced cell death. Treatment of CoCl_2_ dephosphorylated IF1 (5), which might favor the binding of IF1 to c-Myc/PGC1α and prevented their inducing effects on cell death. Overexpression of IF1 appeared to blunt the production of mitochondrial ROS (**Figure 6G**), which may be due to decreased OXPHOS activity and enhanced activity of the permeability transition pore (PTP) (33). Suppression of OXPHOS activity by IF1 overexpression may involve its interactions with c-Myc/PGC1α.

## Discussion

IF1 is a well-known intrinsic inhibitor of F-ATP synthase and plays a role in regulating metabolic phenotypes (1, 6, 9, 10). This study presents another potential mechanism though which IF1 mediates metabolic reprogramming. C-Myc stimulates gene transcription including many metabolic genes, contributing to metabolic reprogramming (17). Thr-58 is a major phosphorylation site in c-Myc, which is mediated by glycogen synthase kinase-3 (GSK-3) (44). Phosphorylation of c-Myc on the Thr-58 site facilitates c-Myc degradation by the ubiquitin pathway (40, 44). IF1 upregulation tended to be accompanied with upregulation of Thr-58 phosphorylated c-Myc (**Figure 1A**). Ablation of IF1 reprogrammed glycolysis to OXPHOS but increased glycolytic capacity and glycolytic reserve (**Figures 1B–H**). IF1 bound to c-Myc and Thr-58 phosphorylated c-Myc in mitochondria (**Figures 2A–C**). Overexpression of c-Myc enhanced cellular glycolytic capacity and suppressed maximal mitochondrial respiration in an IF1-dependent manner (**Figures 2E–M**; **Figure S1**). These observations suggested that IF1 bound to c-Myc and thus promoted the degradation of c-Myc in mitochondria, resulting in the inhibition of mitochondrial gene transcription and mitochondrial biogenesis.

PGC1α synchronizes the mitochondrial and nuclear genomes and thus coordinates mitochondrial biogenesis (23, 24). Activation of PGC1α stimulates mitochondrial biogenesis (25). PGC1α functions as a critical adaptor for maintenance of metabolic balance, and the ratio of c-Myc/PGC1α acts as a main controller of metabolic phenotypes (27). The binding of IF1 to PGC1α caused a dramatic decrease in mitochondrial oxidative phosphorylation (**Figure 4**). c-Myc and PGC1a were localized to mitochondria under mitochondria stress condition, and IF1 was required for this process (**Figure 5**). This phenomenon may be shared with many nuclear proteins including prohibitin and p53 (45). We further performed the cross-linking by DTBP in isolated mitochondria from HEK293T cells, which could be reversibly cleaved by DTT (46), but no interactions were detected, indicating that c-Myc and PGC1a appeared to interact indirectly with IF1. The direct interactions should not be essential for the functional role of c-Myc and PGC1a bound to IF1. Somehow similar, G protein–coupled receptor 35 (GPR35) appeared to interact indirectly with IF1 and was activated in an IF1-depedent manner, which then induced ATP synthase dimerization, which prevented ATP loss upon ischemia (47).

Overwhelming lines of evidence suggest that F-ATP synthase contributes to the regulation and formation of PTP (48–55). The very recent work demonstrates that IF1 plays a role in regulation of the PTP through the interaction with the p53–CypD complex (33). The PTP plays an important role in maintenance of cellular ROS/Ca^2+^ homeostasis. More studies await to investigate that IF1 regulates metabolic rewiring and cell fate through the PTP involving transcription factors and ROS/Ca^2+^ (9, 33, 56).

A sensor of cellular energetic status, is activated by increased ADP/ATP and AMP/ATP ratios (18). The activation of AMPK stabilizes phosphorylation of tumor suppressor protein p53 at Ser-15, resulting in mitochondrial accumulation of p53 (18). Mitochondrial p53 mediates oligomerization of Bak and cell apoptosis (18). The OSCP subunit of F-ATP synthase is identified to be a new partner of mitochondrial matrix p53 (57). Oxidative stress induces the formation of the p53–CypD complex and triggers PTP opening (58). Ablation of IF1 prevents oxidative stress-induced cell death (33). SIRT3 binds to the OSCP subunit of F-ATP synthase in a stress- and pH-dependent manner, which is fundamental in maintaining mitochondrial membrane potential homeostasis in response to mitochondrial stress (59). The upregulated expression levels of both SIRT3 and OSCP may contribute to cell adaption to mitochondrial stress. Ablation of IF1 prevented the protective role of CoCl_2_ in IF1/c-Myc/PGC1α overexpression-induced cell death and also promoted hypoxia-induced cell death (**Figure 6**). These data indicated that bindings of IF1 to c-Myc/PGC1α in regulation of metabolic rewiring were important for cell survival under hypoxic environment. IF1 has been demonstrated to be involved in the development of carcinoma, and attenuates cancer cell sensitivity to chemotherapy (60). In addition to its variabilities in different phenotypes of cancer, IF1 is augmented in many other pathologies including inflammatory myopathies especially dermatomyositis in accordance with metabolic rewiring prone to carcinogenesis (61). Thus, IF1 acts as a novel clinical biomarker of dermatomyositis and a potential metabolic driver of cancer incidence (61). Novel pharmacological inhibitors of IF1 are developed to counteract pathologies (62).

## Materials and methods

### Cell lines and antibodies

HEK293T, HCT116, HeLa, and MIA PaCa-2 cells were supplied by Cell Bank, Chinese Academy of Sciences. Cells were cultured in DMEM medium (high glucose, pyruvate, Gibco, C11995500BT) supplemented with 10% fetal bovine serum (FBS). The primary antibodies are as follows: ATPIF1 polyclonal antibody (12067-1-AP, Proteintech); PGC1α monoclonal antibody (66369-1-Ig, Proteintech); HDAC1 polyclonal antibody (10197-1-AP), ATP5A1 polyclonal antibody (14676-1-AP, Proteintech); ATPB polyclonal antibody (17247-1-AP, Proteintech); ATP5O polyclonal antibody (10994-1-AP, Proteintech); SirT3 (C73E3) rabbit mAb (#2627, Cell Signaling Technology); p-c-Myc (Thr 58) rabbit polyclonal antibody (sc-135647, Santa Cruz Biotechnology); Myc/c-Myc mouse monoclonal antibody (sc-40, Santa Cruz Biotechnology); and Anti-TOMM20 antibody (db50, diagbio).

### Generation of ATPIF1 knockout cell line

The *ATPIF1* knockout cell line was generated using the CRISPR/Cas9 technique (63). Briefly, the specific single guide RNA (sgRNA) was constructed into the lentiviral expression vector for Cas9 and sgRNA. The lentiCRISPR vector was linearized by the BsmBI restriction enzyme (New England Biolabs). The sequences of sgRNA are as follows: ATPIF1 sgRNA-1: 5′-CACCGCAGTGACGGCGTTGGCGGCG-3′ (forward), 3′-AAACCGCCGCCAACGCCGTCACTGC-5′ (reverse); ATPIF1 sgRNA-2: 5′-CACCGTCCAGCAGCAATGGCAGTGA-3′ (forward), 3′-AAACTCACTGCCATTGCTGCTGGAC-5′ (reverse); ATPIF1 sgRNA-3: 5′-CACCGGGCTTGGCGTGTGGGGCGTG-3′ (forward), 3′-AAACCACGCCCCACACGCCAAGCCC-5′ (reverse).

### Immunoblot analysis

Cells were collected by centrifugation at 600 × *g* for 5 min at 4°C, and lysed in RIPA buffer supplemented with protease inhibitors on ice for 30 min. Supernatant was harvested after centrifugation at 12,000 × *g* for 30 min. Protein concentration was determined by BCA assay. The protein solution was supplemented with SDS sample loading buffer followed by SDS-PAGE gel electrophoreses. Proteins were transferred to nitrocellulose membranes followed by membrane blocking with 5% (w/v) nonfat dry milk. The blotted membranes were incubated with primary antibodies overnight at 4°C. The bands were visualized with enhanced chemiluminescence.

### Seahorse analysis of OXPHOS and glycolysis

OXPHOS and glycolysis activities were detected with an Agilent Seahorse XFe24 Analyzer. HCT116 and HeLa cells were seeded in XF24 microplates at 2×10^4^ cells/well in DMEM medium supplemented with 10% FBS. Cells were incubated at 37°C in a 5% CO_2_ humidified incubator for 48 h, or 24 h later, cells were transfected with plasmids and then incubated for 24 h. The culture medium was replaced with Seahorse XF Base Medium (without Phenol Red) supplement with 1 mM pyruvate, 2 mM glutamine, and 10 mM glucose. Cells were incubated at 37°C for 45 min to allow temperature and pH equilibration. Following the baseline measurement, 2 µM oligomycin, 0.25 µM FCCP, 1 µM rotenone plus antimycin A, and 50 mM 2-deoxyglucose (2-DG) were sequentially added to each well. The oxygen consumption rate (OCR) values were subtracted for the rotenone and antimycin-insensitive respiration. The extracellular acidification rate (ECAR) values were subtracted for the 2-DG insensitive glycolysis. The values of OCR and ECAR where indicated were normalized for µg of protein determined by BCA assay.

### Isolated mitochondria from human cultured cells

HEK293T or HeLa cells were collected by centrifugation at 600 × *g* for 5 min and washed once with prechilled PBS buffer. Cell pellet was resuspended in isolation buffer containing 250 mM sucrose, 10 mM Tris-HCl, and 0.1 mM EGTA, pH 7.4, and homogenized with a Potter homogenizer. The supernatant was collected after centrifugation at 600 × *g* for 5 min. The mitochondrial pellet was harvested and washed once with isolation buffer by centrifugation at 8,000 × *g* for 15 min. BCA assay was used to determine mitochondrial protein concentration.

### Expression vectors

cDNA clones of *ATPIF1*, *c-Myc* containing XhoI and EcoRI restriction sites were obtained by a PCR-based method using the whole cDNA of HEK293T as template. The primers used for PCR are as follows: for *ATPIF1*: 5′-ATGCGAATTCATGGCAGTGACGGCGTTGGC-3′ (forward), 3′-ATGCCTCGAGATCATCATGTTTTAGCATTT-5′ (reverse); for *c-Myc*: 5′-CAGTGTGCTGGAATTCTGGATTTTTTTCGGGTAGTGGA-3′ (forward), 3′-CGTAGGGGTACTCGACGCACAAGAGTTCCGTAGC-5′ (reverse). The C terminus HA-tagged pCDNA3.1 vector was linearized by XhoI and EcoRI restriction enzymes (New England Biolabs). The PCR products were cloned into the linearized vector using a ligation high kit (Toyobo). The inserted genes were verified by DNA sequencing.

### Transient transfection

Cells were seeded in a 6-well plate and cultured overnight. Plasmid DNA transfection was achieved by a polyethylenimine (PEI)-mediated transfection method. For one well, a mixture of 2 µg of plasmids, 6 µl of PEI, and 200 µl of free DMEM medium was incubated at room temperature for 15 min, and added to the six-well plate. After 24 h, proteins were extracted for immunoprecipitation or immunoblotting.

### Immunoprecipitation

Cells were solubilized in CoIP lysis buffer containing 50 mM Tris-HCl (pH 7.4), 150 mM NaCl, 1 mM EDTA, 0.5% Nonidet P-40, 10% glycerophosphate, and a cocktail of proteinase inhibitors. The supernatant was collected after a centrifugation at 12,000 × *g* for 20 min at 4°C. Cell lysate was incubated with anti-HA Agarose Beads (Abmart), or incubated with Protein A/G Plus Agarose Beads (Santa Cruz) supplemented with ATPIF1 antibody at 4°C overnight. The beads were washed three times with PBS buffer supplemented with 0.1% NP-40, and boiled in 2× loading buffer for 10 min. Immunoblot analysis was then performed to detect the proteins that were pulled down.

### Immunofluorescence

Cells were seeded in a 12-well dish on sterile 18-mm glass coverslips, cultured overnight, and rinsed three times with PBS for 5 min each. The fixation step was initiated with the addition of 4% paraformaldehyde and incubated at room temperature for 15 min. Aspirated fixative and rinsed three times with PBS for 5 min each. Cells were permeabilized with 0.1% Triton X-100, incubated at room temperature for 10 min, aspirated with Triton X-100, and rinsed three times with PBS for 5 min each. Cells were blocked with 5% bovine serum albumin (BSA) for 1 h at room temperature; aspirated with blocking solution; incubated with 1:100 dilution primary antibodies against IF1 (Proteintech, 12067-1-AP), PGC1α (Proteintech, 66369-1-Ig), c-Myc (Santa Cruz Biotechnology, sc-40), and TOM20 (Proteintech, 11802-1-AP) at 4°C overnight; rinsed three times with PBST buffer (PBS with 0.1% Tween 20) for 5 min each; incubated with DAPI and 1:200 dilution of secondary antibodies (goat anti-mouse IgG Alexa Fluor 488 and goat anti-rabbit IgG Alexa Fluor 647) in 5% BSA at room temperature for 1 h; and rinsed three times with PBST buffer for 5 min each. Fluorescence images were acquired with confocal microscopy (Leica STELLARIS 5).

### Mitochondrial ROS detection

Cells were seeded in a 96-well plate at 1×10^4^ cells/well in DMEM medium supplemented with 10% FBS, and incubated at 37°C in a 5% CO_2_ humidified incubator overnight. Cells were then transfected with empty C terminus HA-tagged pCDNA3.1 vector or *ATPIF1* expressing vector. Cells were cultured for another 24 h to allow the expression of inserted gene. The culture medium was replaced with 5 µM MitoSOX working solution in Hanks’ Balanced Salt Solution (HBSS), incubated at 37°C for 10 min, and rinsed three times with HBSS. Fluorescence images were acquired with a Th4 200 inverted fluorescence microscope (Olympus).

### Cell viability assay

Cells were seeded in 96-well plates at 5×10^3^ cells/well in DMEM medium supplemented with 10% FBS and incubated at 37°C in a 5% CO_2_ humidified incubator overnight. Cells were treated with 0.2 mM CoCl_2_, and transfected with empty C terminus HA-tagged pCDNA3.1 vector or *ATPIF1* expressing vector. Cells were maintained at 37°C in a 5% CO_2_ humidified incubator for 24 h. One hundred microliters of freshly prepared MTT solution (5 mg/ml) was added to each well and incubated at 37°C for 4 h. Then, 100 µl of 10% SDS was added to each well, and OD value at 570 nm was measured with an Infinite 200 PRO plate reader (Tecan).

### Statistical analysis

All the data were presented as mean ± SD of at least three independent experiments, or as representative blots and images. *p*-values indicated in the figures are calculated with GraphPad. One-way ANOVA was used to analyze the difference between more than two groups. Two-way ANOVA was used to determine the interaction between two independent variables. ^*^ *p* < 0.05 was considered statistically significant.

## Data availability statement

The original contributions presented in the study are included in the article/**Supplementary Material**. Further inquiries can be directed to the corresponding author.

## Author contributions

LG: Conceptualization, Methodology, Data curation, Investigation, Data Curation, Software, Validation, Writing and Editing, Visualization, Funding acquisition. ZG: Editing, Funding acquisition. The authors read and approved the final manuscript. All authors contributed to the article and approved the submitted version.
