# Supplementary material for: F-ATP synthase inhibitory factor 1 regulates metabolic reprogramming involving its interaction with c-Myc and PGC1α
Source: Front Oncol. 2023 Jul 3;13:1207603. doi: 10.3389/fonc.2023.1207603 (PMC10352482; doi:10.3389/fonc.2023.1207603)
Supplement: Supplementary file 1 [file DataSheet_1.docx]

Supplementary Material for

F-ATP Synthase Inhibitory Factor 1 Regulates Metabolic Reprogramming Involving its Interaction with c-Myc and PGC1α

**Lishu Guo^1,2,*^, Zhenglong Gu^1^**

From the ^1^Center for Mitochondrial Genetics and Health, Greater Bay Area Institute of Precision Medicine (Guangzhou), Fudan University, Nansha District, Guangzhou 511400, China; ^2^Tongji University Cancer Center, Shanghai Tenth People's Hospital, School of Medicine, Tongji University, Shanghai 200092, China

^*^For correspondence: Lishu Guo, [guolishu@tongji.edu.cn](mailto:guolishu@tongji.edu.cn) or [guolsh15@gmail.com](mailto:guolsh15@gmail.com)

**This PDF file includes:**

SUPPLEMENTARY FIGURE 1


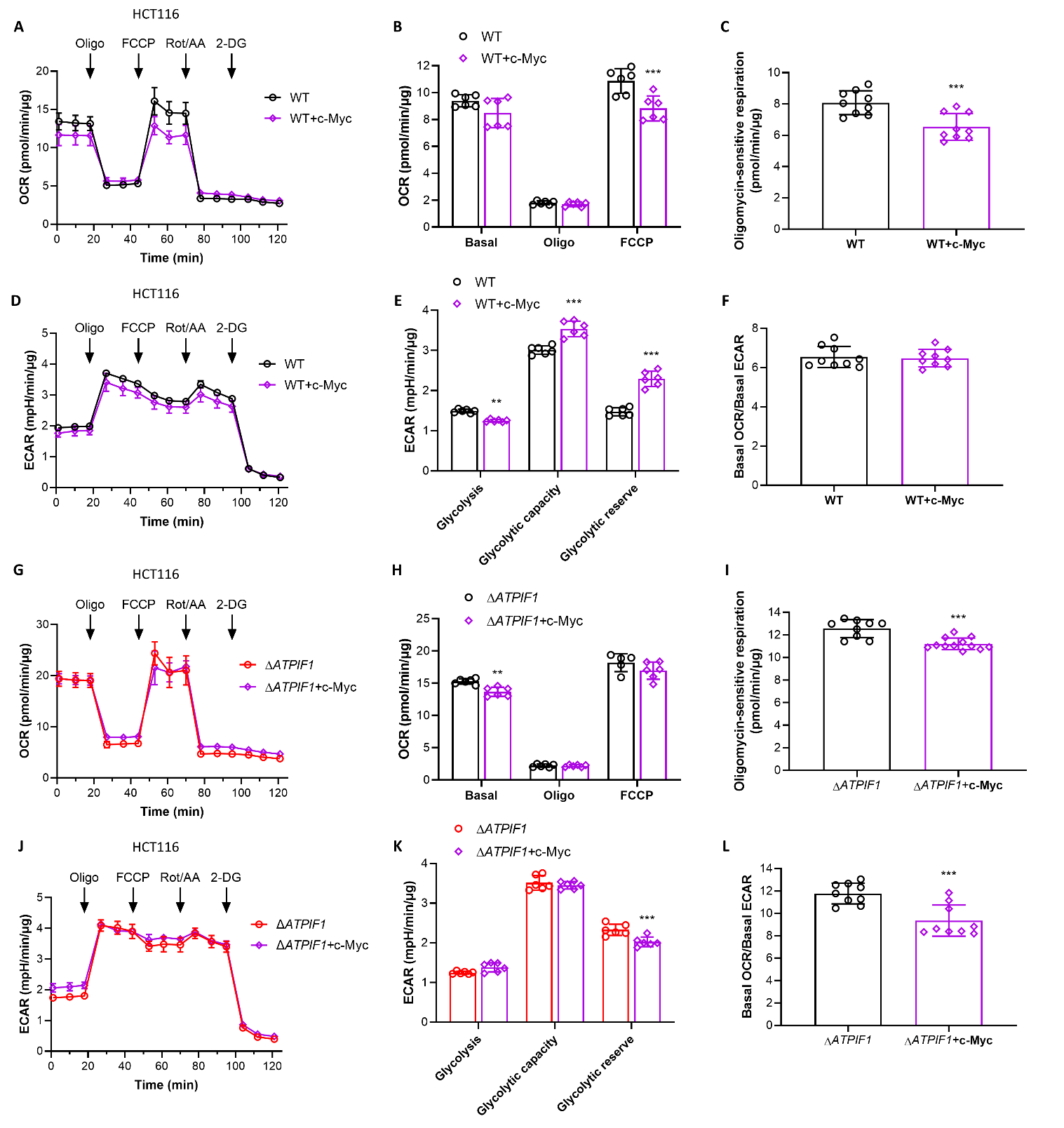


**SUPPLEMENTARY FIGURE 1**

**IF1 is required for the reprogramming of bioenergetic reserve capacity driven by c-Myc.** WT and Δ*ATPIF1* HCT116 cells were transfected with EV or plasmids carrying *c-Myc* and incubated for 24 h. OXPHOS and glycolysis activities were evaluated by Agilent Seahorse XFe24 Analyzer before and after additions of oligomycin (Oligo, 2 µM), FCCP (0.25 µM), rotenone plus antimycin A (Rot/AA, 1 µM), and 2-DG (50 mM). OCR values (pmol/min) and ECAR values (mpH/min) were normalized for protein (µg). *A* and *G,* representative traces of OCR values (pmol/min/µg) in WT (A) and Δ*ATPIF1* (G) HCT116 cells transfected with EV (black trace) and plasmids carrying *c-Myc* (purple trace). *B* and *H,* OCR values (pmol/min/µg) were subtracted for Rot/AA and expressed as mean ± SD. *C* and *I,* oligomycin-sensitive respiration was expressed as mean ± SD. *D* and *J,* representative traces of ECAR values (mpH/min/µg) in WT (D) and Δ*ATPIF1* (J) HCT116 cells transfected with EV (red trace) and plasmids carrying *c-Myc* (purple trace). *E* and *K,* ECAR values (mpH/min/µg) were subtracted for 2-DG and expressed as mean ± SD. ^*^*p* < 0.05 *vs* EV, ^**^*p* < 0.01 *vs* EV, ^***^*p* < 0.001 *vs* EV, two-way ANOVA with Bonferroni post hoc test. *F* and *L,* ratio of basal OCR value and basal ECAR value in WT (F) and Δ*ATPIF1* (L) HCT116 cells. ^***^*p* < 0.001 *vs* EV, two-tailed unpaired Student *t* test.
